# Broadly Protective Monoclonal Antibodies against H3 Influenza Viruses following Sequential Immunization with Different Hemagglutinins

**DOI:** 10.1371/journal.ppat.1000796

**Published:** 2010-02-26

**Authors:** Taia T. Wang, Gene S. Tan, Rong Hai, Natalie Pica, Erin Petersen, Thomas M. Moran, Peter Palese

**Affiliations:** 1 Department of Microbiology Mount Sinai School of Medicine New York, New York, United States of America; 2 Department of Medicine Mount Sinai School of Medicine New York, New York, United States of America; Erasmus Medical Center, Netherlands

## Abstract

As targets of adaptive immunity, influenza viruses are characterized by the fluidity with which they respond to the selective pressure applied by neutralizing antibodies. This mutability of structural determinants of protective immunity is the obstacle in developing universal influenza vaccines. Towards the development of such vaccines and other immune therapies, our studies are designed to identify regions of influenza viruses that are conserved and that mediate virus neutralization. We have specifically focused on viruses of the H3N2 subtype, which have persisted as a principal source of influenza-related morbidity and mortality in humans since the pandemic of 1968. Three monoclonal antibodies have been identified that are broadly-neutralizing against H3 influenza viruses spanning 40 years. The antibodies react with the hemagglutinin glycoprotein and appear to bind in regions that are refractory to the structural variation required for viral escape from neutralization. The antibodies demonstrate therapeutic efficacy in mice against H3N2 virus infection and have potential for use in the treatment of human influenza disease. By mapping the binding region of one antibody, 12D1, we have identified a continuous region of the hemagglutinin that may act as an immunogen to elicit broadly protective immunity to H3 viruses. The anti-H3 monoclonal antibodies were identified after immunization of mice with the hemagglutinin of four different viruses (A/Hong Kong/1/1968, A/Alabama/1/1981, A/Beijing/47/1992, A/Wyoming/3/2003). This immunization schedule was designed to boost B cells specific for conserved regions of the hemagglutinin from distinct antigenic clusters. Importantly, our antibodies are of naturally occurring specificity rather than selected from cloned libraries, demonstrating that broad-spectrum humoral immunity to influenza viruses can be elicited in vivo.

## Author Summary

Influenza viruses remain a formidable public health threat. Because of a dramatic increase in drug resistant strains of influenza viruses and due to the semi-regular emergence of pandemic virus strains, the development of novel antibody-based therapies and influenza vaccine constructs is of great interest. Recently, monoclonal antibodies with broad neutralizing activity against an array of Group 1 influenza viruses (including H5 and H1 subtypes) were identified; studies using these antibodies have expanded our understanding of structural aspects of the viral hemagglutinin, the molecule mediating protective immunity to influenza viruses. We have identified the first broadly neutralizing antibodies against viruses in Group 2—specifically, they are active against H3 influenza viruses spanning 40 years. The antibodies react with the hemagglutinin and appear to bind in regions that are refractory to the structural variation required for viral escape from neutralization. The antibodies demonstrate therapeutic efficacy in mice against H3N2 virus infection and have potential for use in the treatment of human influenza disease. By mapping the binding region of one antibody, 12D1, we have identified a continuous region of the hemagglutinin that may act as an immunogen to elicit an immune response conferring broad protection against H3 viruses.

## Introduction

Under non-pandemic conditions, the global mortality attributed to influenza virus infection is considerable, with 200,000–500,000 associated deaths occurring each year [1]. In the setting of the 1918 influenza pandemic, the global mortality reached 50 million people in one year, equivalent to twice the number of people killed by HIV/AIDS since its emergence almost thirty years ago [2]. Notably, in 1918 and in the current swine-origin influenza virus pandemic, the populations normally considered the fittest are observed to be among the most vulnerable [3],[4].

Four kinds of influenza viruses are circulating in the human population at this time: influenza A viruses of the hemagglutinin H3 and H1 subtypes (H1 viruses are further divided into those of human and swine origin) and influenza B viruses. Influenza A viruses are responsible for the bulk of seasonal disease, with H3 viruses dominating eight of the past twelve influenza seasons in the United States [5]. In 1968, an H3 virus caused one of the three major influenza pandemics of the twentieth century and H3 viruses have persisted since that time as a significant agent of human disease. In addition to humans, H3 influenza viruses commonly infect birds, swine, and horses. It is not known whether H3 viruses will persist as human pathogens or how they may evolve to become more or less virulent in humans.

Immunity to influenza viruses is currently achieved by vaccination with strains representing those predicted to circulate in the coming flu season. In a healthy person, the virus acts as a robust immunogen, eliciting neutralizing serum antibody that protects against influenza disease. Both the humoral and cell-mediated arms of the adaptive system are involved in resolution of active influenza infection, with neutralizing antibody titers correlating with protection *in vivo* [6].

The hemagglutinin glycoprotein is the primary target of antibodies that confer protective immunity to influenza viruses. Antibodies to other influenza proteins likely act in: Fc-receptor mediated uptake of virus particles, antibody-dependent cell cytotoxicity, delay of replication kinetics and, in aggregate, they may contribute to virus neutralization. On a monoclonal level, however, only antibodies specific for the viral hemagglutinin have been shown to block/neutralize infection [7].

Neutralizing monoclonal antibodies (mAbs) act by preventing either of the two functions of the hemagglutinin molecule: virus attachment or virus fusion with the host cell [8]. Antibodies that prevent attachment bind antigenic sites surrounding the receptor binding pocket in the membrane distal HA1 subunit of the hemagglutinin and restrict the association with host cell receptors (sialic acids) [9]. These antibodies drive the outgrowth of antigenic variants, resulting in a continuum of changes in the hemagglutinin structure known as ‘antigenic drift’. Relatively few examples of fusion-inhibiting mAbs are available, but they are most commonly described to interact with the membrane proximal HA2 portion of the hemagglutinin in the region of the fusion peptide [10],[11],[12].

The sixteen subtypes of the influenza hemagglutinin are divided broadly into two phylogenetic groups that correlate with two basic structures taken by the stalk of the molecule [13]. In 1993, mAb C179, an antibody with broad neutralizing activity against viruses in Group 1 (of H1 and H2 subtypes) was described [14]. More recently, several other monoclonal antibodies that neutralize a broad array of Group 1 viruses (including representative H1 and H5 viruses) were identified [11],[12],[15],[16]. These antibodies have consistently been shown to interact with the stalk of the hemagglutinin and neutralize virus by preventing fusion with the host cell. This report constitutes the first description of broadly neutralizing antibodies against viruses in Group 2.

## Results

### Isolation of broadly-reactive anti-H3 mAbs

In order to enhance the production of cross-reactive antibody specificities, we immunized mice by sequential administration with DNA coding for the hemagglutinin from H3 viruses arising approximately 10 years apart: A/Hong Kong/1/1968, A/Alabama/1/1981, A/Beijing/47/1992. Finally, three days prior to fusion, mice were boosted with the H3 virus A/Wyoming/3/2003. By performing the fusion rapidly after virus boost we ensured that only hemagglutinin-specific B cells were present in the spleen at time of fusion. The hemagglutinins chosen were from viruses that arose over several decades, thus representing multiple H3 antigenic clusters [17]. Post-fusion, hybridoma supernatants were screened for the ability to bind A/Hong Kong/1/1968 by western blot or by ELISA and successive rounds of subcloning were performed on positive supernatants until monoclonal hybridoma populations were isolated.

**Figure 1:**
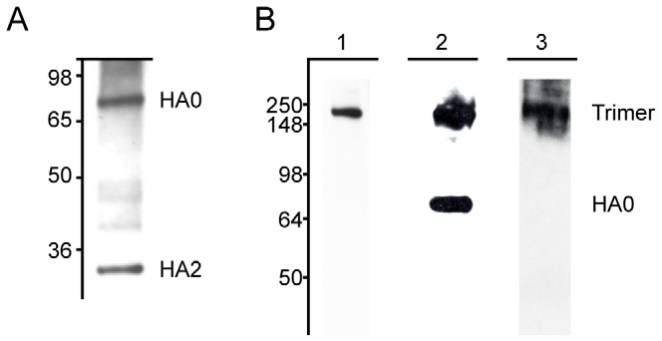
MAbs react with H3 hemagglutinin by western blot. (**A**) MAb 12D1 binds the A/Pan/2007/1999 hemagglutinin within the HA2 subunit. mAbs 7A7 and 39A4 do not react with hemagglutinin under reducing conditions. (**B**) MAbs 7A7, 12D1 and 39A4 react with the A/HK/1/1968 hemagglutinin under non-reducing conditions. MAbs 7A7 (lane 1) and 39A4 (lane 3) bind HA trimer complexes. mAb 12D1 (lane 2) binds HA trimer complexes and HA0.

The immunization schedule we utilized successfully elicited the production of antibodies with broad reactivity against H3 viruses. Approximately 120 clones were isolated that reacted with A/Hong Kong/1/1968; of those, eight mAbs were cross-reactive against all of the H3 hemagglutinins tested. Interestingly, the particular immunization protocol also preferentially elicited the production of antibodies specific for the HA2 subunit of the hemagglutinin. Of the 8 mAbs identified, 5 mAbs react with HA2 and 1 mAb reacts with HA1 by western blot. The remaining 2 mAbs bind conformational epitopes present in the HA trimer as detected by western blot of purified H3 virus proteins separated under non-reducing gel conditions. All mAbs were reactive in a purified H3 virus ELISA. Three of the mAbs, 7A7, 12D1, 39A4, had the highest activity by ELISA and were selected for thorough characterization (Table 1, Figure 1).

**Table 1:** Pattern of reactivity of anti-H3 mAbs.

	Isotype	ELISA	WB	HI
7A7	IgG2b	+	Trimer	−
12D1	IgG1	+	HA2	−
39A4	IgG2a	+	Trimer	−
62F11	IgG2a	+	HA2	−
36A7	IgG2b	+	HA2	−
66A6	IgG1	+	HA1	−
49E12	IgG2b	+	HA2	−
21D12	IgG1	+	HA2	−

All mAbs have activity by ELISA and all mAbs react by western blot under reducing conditions except mAbs 7A7 and 39A4 that react with the HA trimer under non-reducing conditions. All mAbs are negative for hemagglutination inhibition (HI) activity at 50ug/ml.

**Figure 2:**
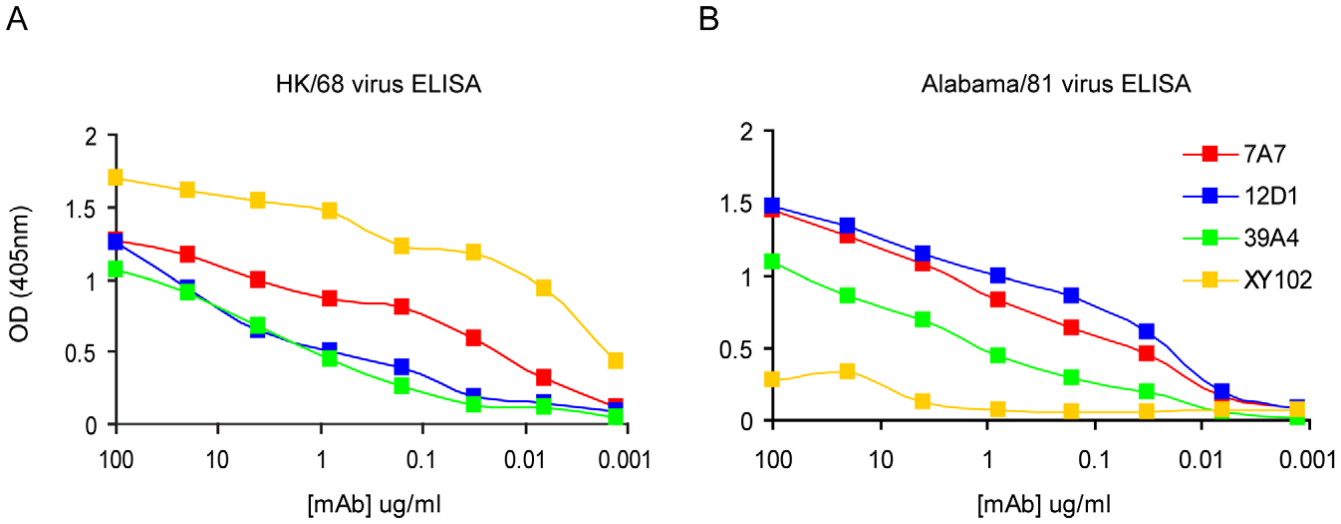
Reactivity of anti-H3 mAbs by ELISA. (**A**) mAbs react with purified A/HK/1968 (H3) virus. (**B**) mAbs react with purified A/Alabama/1981 (H3) virus. mAb XY102 is specific for the hemagglutinin of A/HK/1968 virus.

Antibodies 7A7, 12D1 and 39A4 react by ELISA with purified A/Alabama/1/1981 and purified A/Hong Kong/1/1968 viruses (Figure 2). MAb XY102 is specific for the hemagglutinin of A/Hong Kong/1/1968 virus [18]. MAbs 7A7, 12D1 and 39A4 show broad reactivity by immunofluorescence against cells infected with all H3 viruses spanning 40 drift years. MAbs 7A7 and 39A4 also react by immunofluorescence with other influenza A viruses chosen at random, including representative H1, H2 and equine H3 viruses (Table 2).

**Table 2:** Reactivity of mAbs at 5ug/ml by immunofluorescence against MDCK cells infected with a panel of randomly chosen viruses.

Virus	Subtype	7A7	12D1	39A4	10C4	XY102
HK/68	H3	+	+	+	−	+
AL/81	H3	+	+	+	−	−
BJ/92	H3	+	+	+	−	−
WI/05	H3	+	+	+	−	−
BR/07	H3	+	+	+	−	−
NY/08	H3	+	+	+	−	−
TX/91	H1	+	−	+	+	−
FM/47	H1	+	−	+	−	−
AA/60	H2	+	−	+	−	−
Equine/KY/02	H3	+	−	+	−	−

MAb XY102 was generated by immunization with A/HK/1968 (H3) virus and mAb 10C4 was generated by immunization with A/TX/1991 (H1) virus.

### Anti-H3 mAbs neutralize H3 viruses spanning 40 drift years

**Figure 3:**
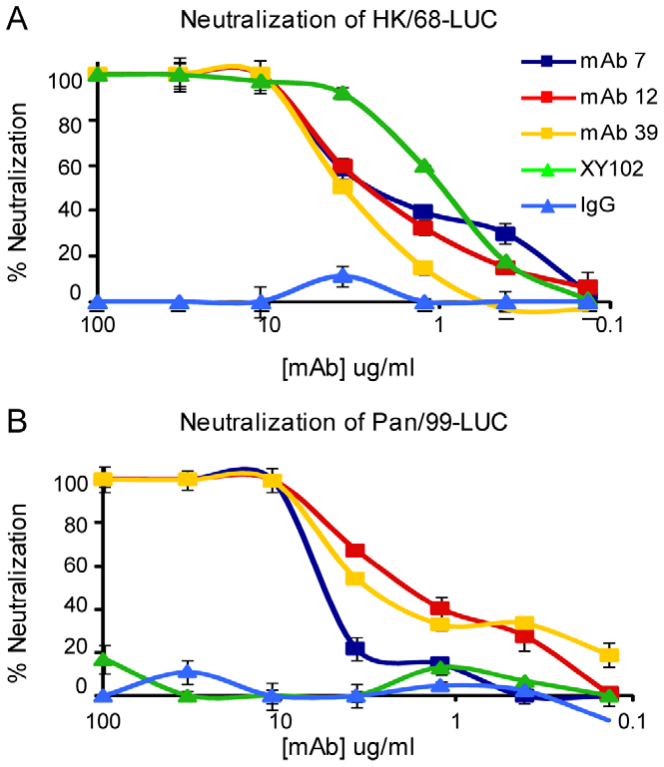
Anti-H3 mabs in microneutralization assay. Neutralization of virus expressing the HA from either (**A**) A/Hong Kong/1/1968 virus or (**B**) A/Panama/2007/1999 virus. mAb XY102 is specific for A/HK/1968 virus. Purified mouse IgG was used for the negative control.

The anti-H3 mAbs were first evaluated for their ability to neutralize H3 influenza viruses by microneutralization assay. Viruses used in this assay contain a gene segment coding for firefly luciferase in place of the viral hemagglutinin; a hemagglutinin is present on the viral envelope due to propagation of virus in cells stably expressing a particular H3 hemagglutinin protein (see methods). Luciferase viruses were generated that express the hemagglutinin of A/HK/1968 or A/Panama/99 viruses. Neutralization of viruses by anti-H3 mAbs was determined based on luciferase activity after single-cycle replication; mAbs 7A7, 12D1 and 39A4 were determined to neutralize the hemagglutinin of both A/HK/1968 and A/Pan/99 (Figure 3).

**Figure 4:**
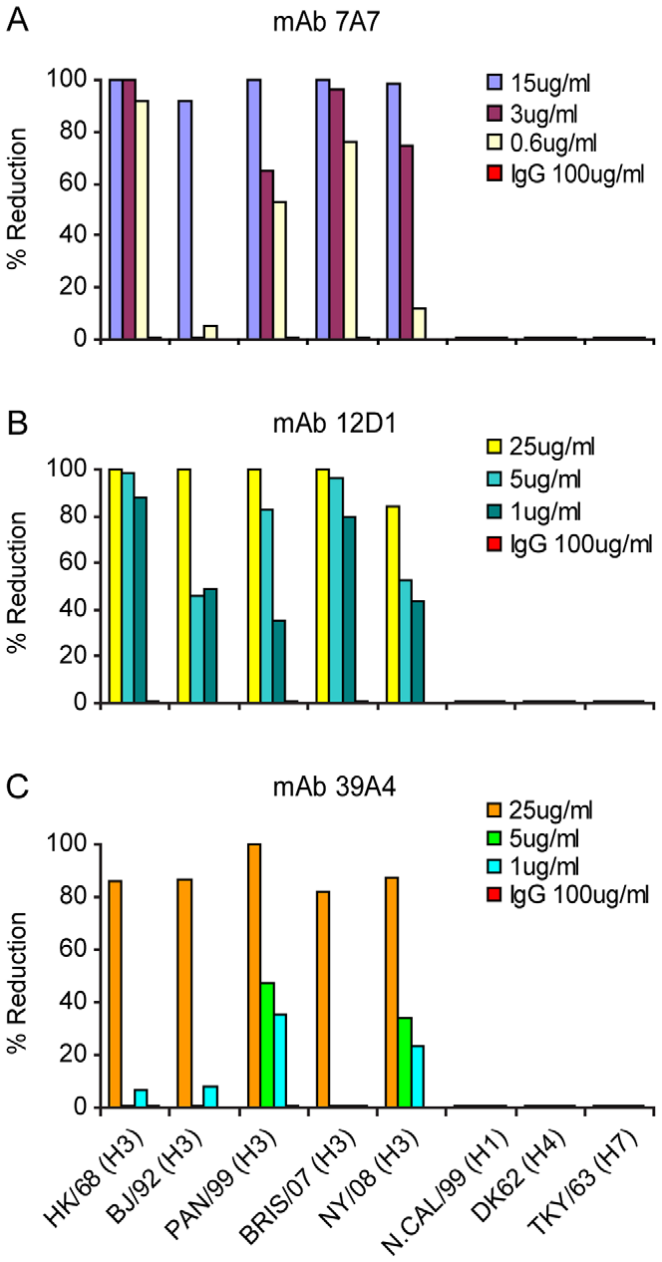
Activity of anti-H3 mabs in plaque reduction assay on MDCK cells. mAb 7A7 (**A**), 12D1 (**B**) and 39A4 (**C**) neutralize all H3 viruses tested by plaque reduction assay but not representative H1, H4 or H7 viruses. Purified mouse IgG was used for the negative control. The plaque reduction assays were performed multiple times and with each new antibody preparation. Data shown are from a single representative experiment.

Next, we evaluated neutralization activity by plaque reduction assay. The anti-H3 mAbs were able to prevent infection (not simply reduce plaque size) of Madin Darby canine kidney cells by H3 viruses arising over 40 drift years: A/HK/1968, A/BJ/1992, A/Pan/99, A/Bris/07, A/NY/08 (Figure 4). We tested 7A7, 12D1 and 39A4 against representative H4 and H7 viruses (Group 2) as well as an H1 virus (Group 1) and found that they did not neutralize the non-H3 subtype viruses (Figure 4).

### Anti-H3 mAbs in the treatment of influenza in mice

**Figure 5:**
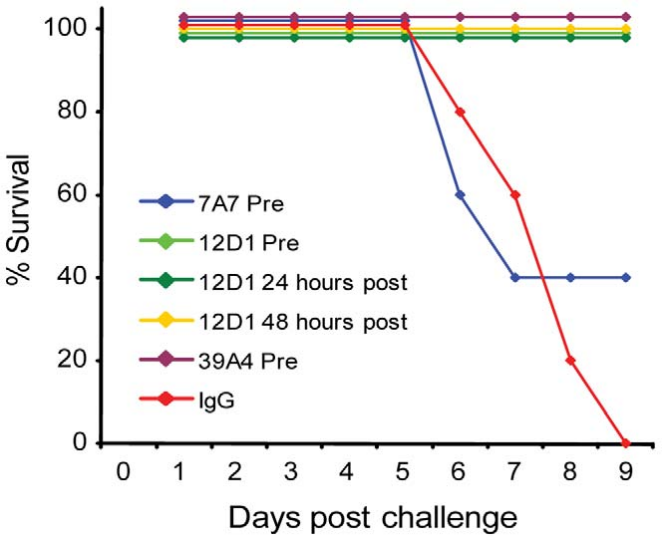
Anti-H3 mAbs protect against H3 virus in vivo. Mice were given 30mg/kg mAb 7A7, 12D1, 39A4 or isotype control by intraperitoneal injection 1 hour prior, 24 hours post (12D1 only) or 48 hours post (12D1 only) challenge with H3N2 reassortant virus (HK68/PR8). N = 5 per group.

The three mAbs were tested *in vivo* for use as passive transfer therapies in disease caused by H3 virus infection. Mice were given 30mg/kg mAb intraperitoneally either 1 hour before, 24 hours post or 48 hours post challenge with 10 mouse LD_50_ reassortant H3 virus (the A/HK/68 reassortant virus contains the six non-hemagglutinin, non-neuraminidase segments from the mouse-adapted A/PR/8 virus). Mice were weighed daily and were sacrificed if they reached 75% of their starting weight. Treatment of mice with mAb 12D1 either prophylactically or therapeutically was 100% protective. mAb 39A4 was evaluated for efficacy by prophylactic treatment and was similarly 100% protective *in vivo*. Mice treated prophylactically with mAb 7A7 were only 40% protected against the A/HK/68 reassortant virus (Figure 5).

**Figure 6:**
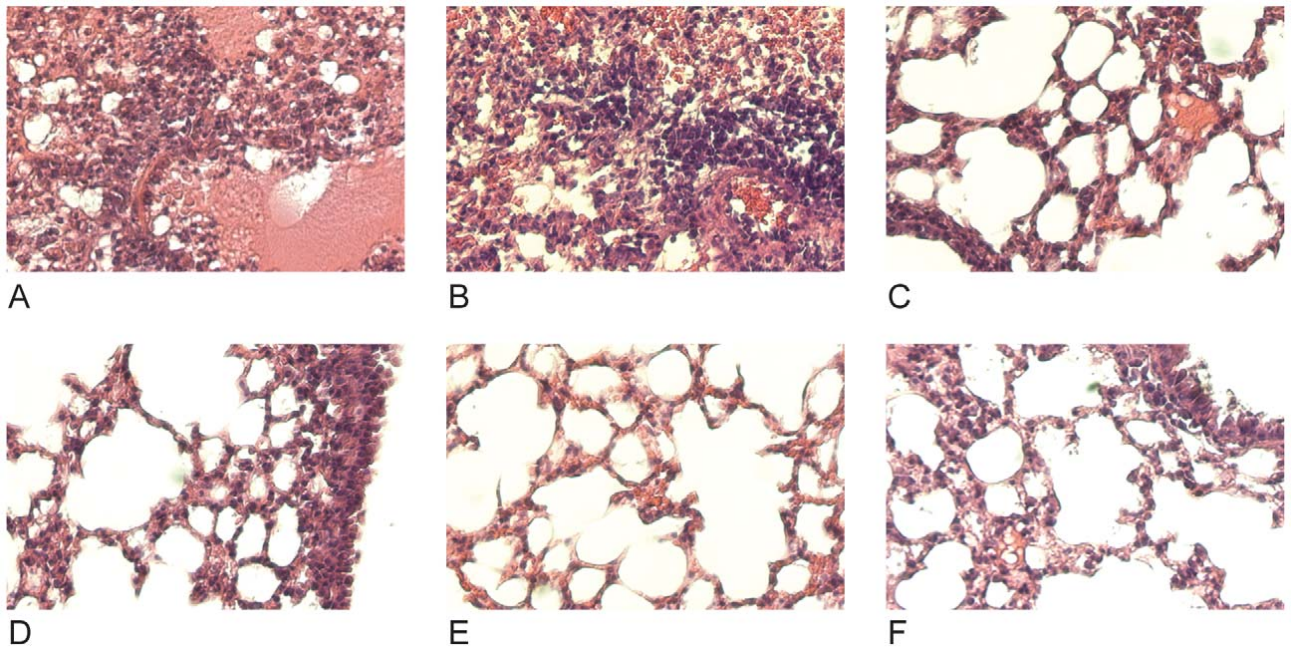
Treatment with anti-H3 mAbs diminishes lung damage associated with viral pneumonia caused by HK68/PR8 reassortant virus. (**A,B**) Untreated (**C,D**) mice treated with mAb 39A4 (**E,F**) mice treated with mAb 12D1. 40× magnification.

Next, the effect of prophylactic treatment with mAb 12D1 or 39A4 on lung damage caused by H3 viral pneumonia was assessed by histologic evaluation of tissue taken 4 days post infection with the A/HK/68 reassortant virus. Without treatment, lungs showed degenerative changes with focal hemorrhaging, dense neutrophilic infiltrates and diffuse alveolar damage with edema. Treatment with either anti-H3 mAb significantly diminished pathologic changes (Figure 6).

**Figure 7:**
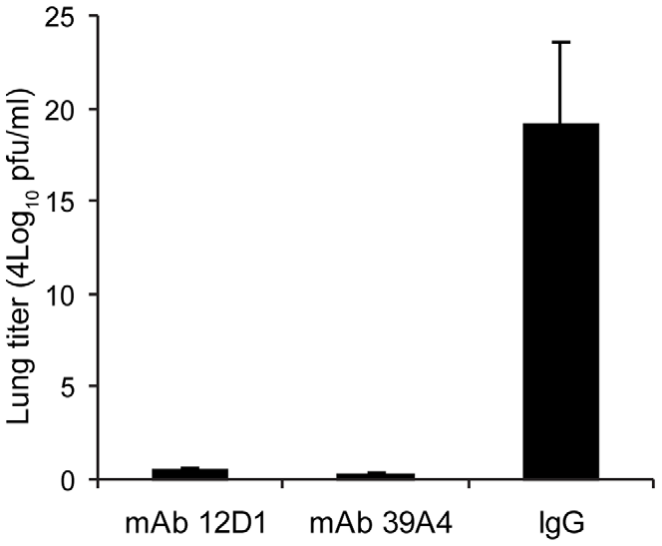
Anti-H3 mAbs protect against replication of H3 virus in lungs. Mice were given 30mg/kg mAb 12D1, 39A4 or isotype control by intraperitoneal injection 1 hour prior to infection with A/Georgia/1981 virus. Data represent lung titers from groups of 5 mice, 2 days post infection.

Having demonstrated protective activity *in vivo* against the A/HK/68 reassortant virus we sought to evaluate cross-protection mediated by mAbs 12D1 and 39A4 against a second H3 virus, A/Georgia/1981. MAbs 12D1 and 39A4 were administered as described above to BALB/c mice one hour prior to infection. Mice were then infected intranasally with 2700 pfu A/Georgia/1981 and lung titers were evaluated two days post infection. The anti-H3 mAbs were found to reduce lung titers by 97.75% (12D1) or 99.03% (39A4) (Figure 7).

### Anti-H3 mAbs act by inhibiting viral fusion

In order to determine the mechanism of virus neutralization by our anti-H3 mAbs, we first looked at the ability of the mAbs to inhibit virus hemagglutination of chicken red blood cells. We found that none of the three mAbs had hemagglutination inhibition activity, suggesting that the mAbs did not act by obstructing the binding of virus to the host-cell.

**Figure 8:**
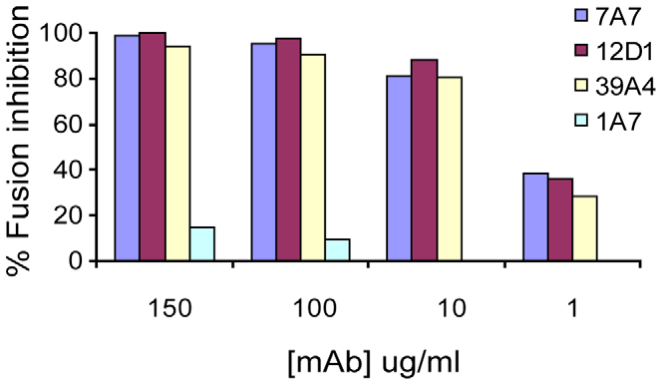
Red blood cell fusion assay. Anti-H3 mabs inhibit low-pH induced fusion of HK/68 hemagglutinin with chicken red blood cells. All mAbs are negative for hemagglutinin-inhibition activity. MAb 1A7 is specific for influenza virus NS1 protein.

Next, we tested the effect of the anti-H3 mabs on virus fusion. MAbs 7A7, 12D1 and 39A4 were determined to inhibit the low-pH fusion of A/HK/1968 virus with chicken red blood cells by at least 80% at 10ug/ml (Figure 8).

### Binding epitope of mab 12D1

Finally, we aimed to identify the region of the H3 hemagglutinin that might elicit antibodies with fine specificities mirroring those of 12D1 or 39A4. Sixteen passages of A/HK/1968 virus in the presence of the anti-H3 mAbs 12D1 or 39A4 did not yield escape variants that might have assisted in identification of the binding epitopes. Also, the hemagglutinin of six plaques present after incubation of A/HK/1968 virus with 50ug/ml mAb 12D1 or 39A4 in a plaque assay was sequenced and we were surprised to find no changes from the wild-type hemagglutinin.

**Figure 9:**
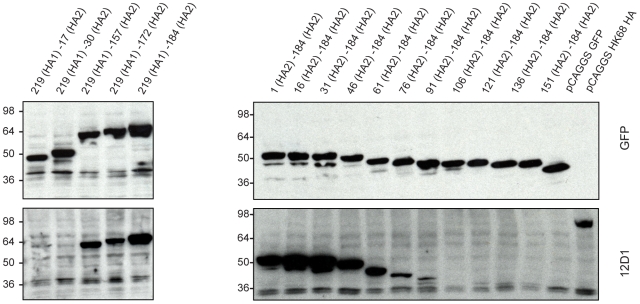
MAb 12D1 reacts by western blot with hemagglutinin truncation mutants. 12D1 makes dominant contacts with the HA2 subunit in the region of amino acids 30 to 106 (H3 numbering[21]). Diminished 12D1 binding without diminished GFP expression in the HA2 76–184 and HA2 91–184 truncations along with loss of binding with the HA2 106–184 truncation suggests that the binding epitope lies in the region from amino acids HA2 76–106. These 30 amino acids fall within the membrane distal half of the long alpha-helix of HA2.

**Figure 10:**
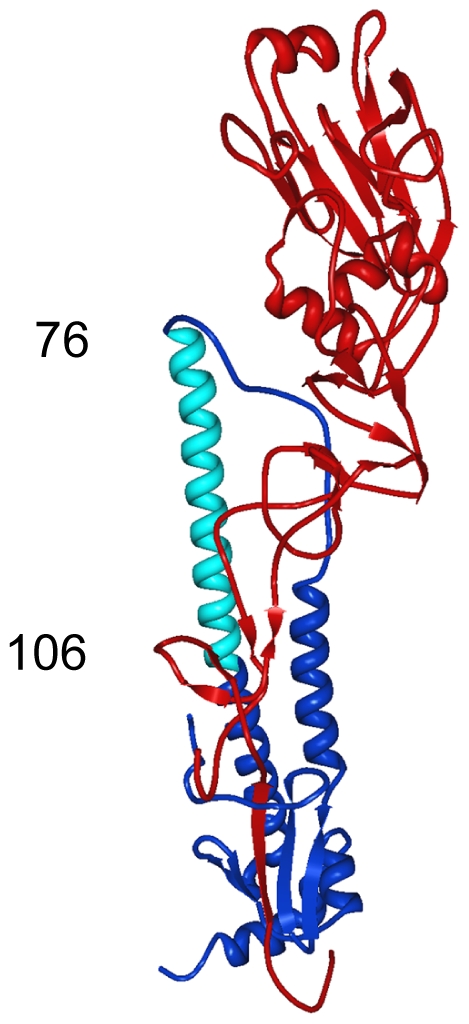
Region of dominant contacts between anti-H3 mab 12D1 and HA2. mAb 12D1 makes contact in the HA2 region (blue) of the viral hemagglutinin. Evaluation of binding to hemaggluinin truncation mutants demonstrates binding within the region of amino acids 76–106 of HA2 (light blue).

Because mAb 12D1 mediates protection against influenza disease *in vivo* and reacts with a continuous epitope of the viral hemagglutinin (no trimeric structure required), as evidenced by reactivity with the denatured hemagglutinin monomer by western blot (Figure 1), we focused on identification of the 12D1 binding epitope. Hemagglutinin truncation mutants consisting of hemagglutinin segments of varying length fused to GFP were generated. GFP expression was utilized to assess expression of the constructs in transfected 293T cells. By analysis of the truncation mutants, it was determined that the 12D1 paratope makes dominant interactions with the HA2 subunit in the region of amino acids 30–106. Diminished 12D1 binding without diminished GFP expression in the 76–184 and 91–184 truncations along with loss of binding with the 106–184 truncation suggested that 12D1 binding is dependent on contacts with amino acids in the HA2 76–106 region (Figure 9). These 30 amino acids fall within the membrane distal half of the long alpha-helix of HA2 (Figure 10). The 12D1 paratope may have additional contacts with amino acids outside of this region (in HA1 or HA2) that are not required for binding by western blot.

## Discussion

For this study, we developed an immunization schedule that elicited broadly-neutralizing antibodies against H3 influenza viruses *in vivo*. The finding that such antibody specificities can be elicited by vaccination of mice suggests that with the proper immunogen(s) and vaccination protocol, such a response might also be elicited in humans. Several recent studies describe antibodies isolated from human phage display libraries that have cross-neutralizing activity against Group 1 influenza viruses [11],[12],[15],[16]. Mabs isolated from human display libraries have proved extremely useful in the characterization of structural epitopes that mediate heterosubtypic neutralization. Caveats to this methodology exist, however, since the diversity of combinatorial display libraries is typically orders of magnitude greater than the diversity of the true human variable region repertoire [19]. Additionally, phage display libraries are generated by random combination of immunoglobulin VH and VL genes and are therefore not restricted, as the *in vivo* repertoire is, by mechanisms regulating the production of auto-reactive specificities.

Until now, broadly neutralizing antibodies reactive with H3 viruses have not been described. Interestingly, mAbs 7A7 and 39A4 react by immunofluorescence with the hemagglutinin of multiple subtypes, though neutralizing activity appears to be limited to H3 viruses. Binding by these mAbs to other subtypes may be of relatively low avidity such that they no longer mediate neutralization, or, they may simply bind an epitope of non-H3 hemagglutinins that does not mediate neutralization. The identification of anti-H3 mAbs 12D1 and 39A4 complements recent works describing antibodies F10 and CR6261 that neutralize an array of Group 1 viruses [11],[12],[16]. One might envision a passive transfer therapy consisting of multiple broadly neutralizing mAbs for general use against pandemic and seasonal influenza virus strains. With the increasing resistance of influenza virus isolates to available anti-viral drugs, such an antibody cocktail could be of great value in severe disease.

Mouse monoclonal antibodies such as the anti-H3 mabs described herein are commonly used in the development of therapeutic antibodies for use in humans. Once characterized, rodent antibodies are readily humanized by methods typically involving grafting of non-human complimentary determining regions into appropriate human frameworks followed by cloning of variable region segments into complete human immunoglobulin constructs [20].

The fact that escape mutants were not selected after multiple passages of virus in the presence of anti-H3 mabs 12D1 and 39A4 is intriguing. Sui et al. reported that they were similarly unable to isolate escape mutants using their fusion-inhibiting mAb F10 [11]. MAb F10 makes multiple interactions with the hydrophobic pocket of the hemagglutinin including with the fusion peptide itself and prevents the low-pH triggered conformation change required for fusion. Considering the rigid structural and electrostatic requirements involved in membrane fusion, the hemagglutinin might not readily accommodate mutations at the F10 binding epitope. Anti-H3 mAb 39A4 binds a conformational epitope of the hemagglutinin trimer; the region of binding may bridge two monomers, therefore interacting with two different portions/faces of each monomer. A mutation at one region of contact (that does not affect trimer formation) may not be sufficient to ablate 39A4 binding. Anti-H3 mAb 12D1 likely binds within the long alpha-helix of HA2. This region may not accommodate changes that would affect 12D1 binding due to required secondary helix structure and specific van der Waals interactions that stabilize the hemagglutinin trimer [21]. Generally, mutations in the stalk of the hemagglutinin are more likely to affect the architecture of the entire molecule than are mutations in the classical antigenic sites [9].

The development of HA2-based vaccine constructs is of significant interest given recent reports of anti-HA2 mAbs with broad neutralizing activity against influenza viruses. Original studies of immunogens consisting of virus particles lacking the HA1 subunit demonstrated that design of an effective construct, however, will likely not be straightforward [22]. This is in large part due to the difficulty involved in maintaining the native configuration of the hemagglutinin stalk, which has complex tertiary structure and incorporates a portion of HA1 in addition to the HA2 subunit. Recent reports of mAbs with broad neutralizing activity against influenza viruses that are not active by western blot and that make contacts with amino acids in both HA1 and HA2 underscore the importance of maintaining non-contiguous epitopes in HA2 vaccine contructs [12],[16],[20].

In contrast to these mAbs, anti-H3 mAb 12D1 does not rely on a structural/non-contiguous epitope of the hemagglutinin stalk for binding. The observation that 12D1 makes dominant contacts within a continuous segment of the HA2 subunit suggests the design of an immunogen, perhaps consisting of that HA2 segment coupled to a carrier protein, that would direct an immune response to the region. The identified region, HA2 76–106, is 100% conserved between the H3 viruses used in this study and all other H3 viruses that we have examined. In contrast, the H1 viruses A/New Caledonia/20/99 and A/PR/8/34 share only 56.7% identity with the equivalent region in the H3 hemagglutinin. A vaccine construct incorporating this region, therefore, would likely not provide protection against H1 influenza viruses. This study and other structural studies [11],[12],[14] of the influenza hemagglutinin provide groundwork for the design of novel vaccine constructs aimed at providing broad-spectrum immunity to influenza viruses.

## Materials and Methods

### Animals

6 week old female BALB/c mice from Jackson Laboratory were used for all experiments. All animal procedures performed in this study are in accordance with Institutional Animal Care and Use Committee (IACUC) guidelines, and have been approved by the IACUC of Mount Sinai School of Medicine.

### Viruses and cells

Madin Darby canine kidney cells from ATCC were used for all cell based assays. Cells were maintained in minimum essential medium supplemented with 10% fetal bovine serum, and 100 units/ml of penicillin-100 µg/ml of streptomycin. All viruses were propagated in eggs. Viruses used in various studies: A/Hong Kong/1/1968 (HK/68) (H3), A/Alabama/1/1981 (AL/81) (H3), A/Georgia/1981 (H3), A/Beijing/47/1992 (BJ/92) (H3), A/Wyoming/3/2003 (H3), A/Wisconsin/67/2005 (WI/05) (H3), A/Brisbane/10/2007 (BR/07) (H3), A/New York/2008 (NY08) (H3), A/Texas/36/1991 (TX/91) (H1), A/New Caledonia/20/99 (N.Cal/99) (H1), A/Duck/England/1962 (Dk/62) (H4), A/Turkey/England/1963 (Tky/63) (H7), A/Equine/Kentucky/2002 (e/KY/02) (H3), A/Ann Arbor/6/1960 (AA/60) (H2), A/Fort Monmouth/1/1947 (FM/47) (H1). Purified virus was prepared by high speed centrifugation (43,000 rpm, 1 hour) of allantoic fluid through a 20% sucrose cushion.

### Antibody preparations

Hybridoma supernatants were used for screening of mAbs for reactivity by enzyme-linked immunosorbent assay (ELISA) and by western blot. For other assays, purified monoclonal antibody or ascites preparations treated with receptor-destroying enzyme [23] were used. RDE –treated ascites was used for measurement of binding by ELISA, microneutralization, plaque reducion and fusion assays. Antibodies were purified by methods previously described [24]. Because of differences in isotypes, Protein A-agarose (Roche) was used for purification of mAbs 7A7 and 39A4 while protein G-agarose (Roche) was used for purification of mAb 12D1.

### Immunization of mice and hybridoma production

6-week old BALB/c mice were immunized with DNA constructs coding for the open-reading frame of influenza virus hemagglutinin in the pCAGGS plasmid [25]. Individual immunizations were given intramuscularly, 3-weeks apart and consisted of 100ug DNA in 100ul PBS. Hemagglutinins utilized in the immunization schedule were cloned from the following parental viruses - primary immunization: A/Hong Kong/1/1968, secondary immunization: A/Alabama/1/1981, tertiary immunization: A/Beijing/47/1992 HA. Three days prior to fusion, mice were boosted with 50ug purified A/Wyoming/3/2003 virus. B cell hybridomas were produced by methods previously described [26],[27].

### Screening of hybridoma supernatants

Hybridoma supernatants were screened by blot and by ELISA for reactivity with A/Hong Kong/1/1968 virus. For the ELISA, direct binding to wells coated with 5ug/ml purified virus, 50ul per well was assessed. For the blot assay, 10ug purified virus was adsorbed onto nitrocellulose strips and individual strips were incubated with hybridoma supernatants. For the ELISA and blot assays, binding of antibody to virus was detected using goat anti-mouse γ-chain horse radish peroxidase secondary antibody (SouthernBiotech, Birmingham, Al). All wells that had activity in either assay against A/Hong Kong/1/1968 virus were subcloned repeatedly to ensure the monoclonality of the hybridoma populations.

### Western blots

Blots were produced by methods previously described [28]. Samples were boiled for 5 minutes at 100°C in loading buffer containing SDS and 0.6M DTT. SDS migration buffer was used for electrophoresis. For non-reducing gel conditions samples were prepared in loading buffer with SDS but without reducing agent and were not boiled.

### Immunofluorescence test

MDCK cells were infected with virus at a multiplicity of infection of 1 and incubated for 6 hours at 37°C. Infected and uninfected cells were incubated with 1ug/ml mAb for 1 hour at room temperature. Goat anti-mouse fluorescein conjugate (SouthernBiotech) was used for detection of mAb binding.

### Microneutralization assay

Two stable cell lines were generated that expressed the HA of A/Hong Kong/1/1968 virus or A/Panama/2007/1999 virus. Pseudotyped viruses expressing the HA of either cell line were generated by infection of cells with a virus that carries seven segments from A/WSN/33 virus (all except the HA segment) and one segment encoding Renilla luciferase. Pseudotyped viruses expressing the HA of A/Hong Kong/1/1968 virus or A/Panama/2007/1999 virus were used as the neutralization target. Viruses were incubated with mAb at room temperature for 30 minutes, rocking. Purified polyclonal mouse IgG (Invitrogen) was used for the negative control. The mixture containing virus and mAb was then transferred to wells of a 96-well plate seeded to confluency with MDCK cells and incubated for 12 hours at 37°C. Individual determinants were performed in triplicate. After incubation, luciferase activity in cell-lysates was measured as a read-out of virus infection (Renilla luciferase assay system, Promega).

### Plaque reduction assay

Antibody and virus (∼50 pfu/well) were co-incubated at room temperature for 30 minutes, rocking. 6 well plates seeded with MDCK cells were washed once with PBS and 200ul of virus and mAb was added to each well then incubated for 20 minutes, 37°C. Virus with mAb was aspirated from cells and an agar overlay containing antibody was added to each well. Plates were incubated for 3 days, 37°C and plaques were counted by crystal violet staining. Purified mouse IgG (Invitrogen) was used for the negative control.

### Passive transfer experiments

Before infection, mice were anesthetized by intraperitoneal administration of a ketamine (75 mg/kg of body weight)/xylazine (15 mg/kg of body weight) mixture. 6 week old BALB/c mice were given 30mg/kg mAb intraperitoneally either one hour before, 24 hours after or 48 hours after challenge with 10 LD_50_ A/Hong Kong/1/1968, A/PR/8/34 reassortant virus or 2700 pfu A/Georgia/1981 virus (lung titer experiment). Purified mouse IgG (Invitrogen) was used for the negative control. Virus was suspended in PBS and administered intranasally in 50ul (25ul per nostril). Mice were weighed daily and sacrificed if they fell to 75% of starting weight. For the lung titer experiment, mouse lungs were harvested 4 days post infection with A/Georgia/1981 and virus titers in lung homogenates were determined by plaque assay. For histologic evaluation of lung damage, lungs were harvested 4 days post infection with A/Hong Kong/1/1968 - A/PR/8/34 reassortant virus. Tissues were imbedded in paraffin and sections were stained with hematoxylin and eosin.

### Hemagglutinin inhibition assay and fusion assay

MAbs were tested in a standard hemagglutination inhibition assay [29] using chicken red blood cells and A/Hong Kong/1/1968 virus. For the red blood cell fusion assay, virus was incubated with chicken red blood cells (2% final red cell concentration) on ice for 10 minutes. Dilutions of antibody were added and samples were incubated on ice for 30 minutes. Sodium citrate buffer, pH 4.6 was then added to bring the final pH to 5.0 and samples were incubated for 30 minutes at room temperature. Samples were centrifuged for 3 minutes at 3000rpm to pellet red blood cells and supernatants were then transferred to an ELISA plate for determination of NADPH content by optical density measurement (340nm). NADPH was present in the supernatant as a function of fusion-induced red blood cell lysis.

### Hemagglutinin truncation mutants

DNA constructs were generated in the pCAGGS plasmid that coded for truncations of the A/HK/1/68 virus hemaggluinin fused to green fluorescent protein. All constructs were sequenced and confirmed. 293T cells were then transfected using Lipofectamine 2000 (Invitrogen, Inc.) with the various pCAGGS encoding the HA-GFP fusion gene. Cell lysates were resolved in a 4–20% Tris-HCl SDS-PAGE gel (Bio-Rad Laboratories) and proteins were blotted onto a Protran nitrocellulose membrane (Whatman). GFP and truncated HA fragments were detected using rabbit anti-GFP (Santa Cruz Biotechnology, Inc.) and anti-H3 mAb 12D1 respectively. Secondary antibodies were anti-rabbit IgG HRP (Dako) and anti-mouse Ig HRP(GE Healthcare).
